# Safety and Efficacy of Endoscopic Derotation in Colonic Volvulus Occlusion: Systematic Review and Meta-Analysis

**DOI:** 10.3390/jcm15031190

**Published:** 2026-02-03

**Authors:** Filippo Sabatini, Luca Properzi, Gabriele Marinozzi, Gabrio Bassotti, Bruno Cirillo, Gioia Brachini, Francesco Brucchi, Sara Lauricella, Alberto Santoro, Matteo Matteucci, Antonia Rizzuto, Roberto Cirocchi

**Affiliations:** 1 Dipartimento di Chirurgia Generale e Digestiva, Università degli Studi di Perugia, Ospedale “Santa Maria”, 05100 Terni, Italy; 2 Dipartimento di Endoscopia Digestiva, Ospedale “Santa Maria”, 05100 Terni, Italy; 3 Dipartimento di Scienze Onco-Emato-Gastroenterologiche, Università degli Studi di Perugia, 06123 Perugia, Italy; gabrio.bassotti@unipg.it; 4 Department of Surgery, Sapienza University, Viale del Policlinico 155, 00161 Rome, Italygioia.brachini@uniroma1.it (G.B.); 5 Department of Medicine and Surgery, University of Milan, 20122 Milan, Italy; 6 Colorectal Surgery Division, Department of Surgery, Fondazione IRCCS Istituto Nazionale Dei Tumori, 20133 Milan, Italy; 7 Department of Surgical Sciences, Sapienza University of Rome, 00185 Rome, Italy; 8 Department of Medical and Surgical Sciences, Magna Græcia University, 88100 Catanzaro, Italy

**Keywords:** sigmoid volvulus, endoscopic detorsion, recurrence, meta-analysis

## Abstract

**Background:** Sigmoid volvulus is a time-critical cause of large-bowel obstruction. While endoscopic detorsion (ED) is the primary intervention for rapid decompression and the assessment of mucosal viability, reported success, recurrence, and mortality rates vary significantly across the literature, complicating evidence-based clinical decision-making. **Methods:** A systematic review and meta-analysis were conducted following PRISMA guidelines (protocol submitted to PROSPERO). MEDLINE/PubMed and Embase were searched from inception to 20 October 2025, supplemented by manual reference screening. We included original prospective or retrospective studies (n ≥ 5) reporting outcomes after ED for sigmoid volvulus, specifically technical success, post-ED recurrence, or mortality. Pooled proportions were estimated using a DerSimonian–Laird random-effects model on the logit scale, with heterogeneity quantified using I^2^ statistics. Administrative database studies were summarized descriptively and excluded from the quantitative synthesis to minimize selection bias. **Results:** Nineteen studies (2004–2025) met the inclusion criteria from an initial 890 records. Fifteen studies (n = 1738) contributed to the analysis of technical success, yielding a pooled estimate of 80.0% (95% CI: 75.0–83.0%; I^2^ = 87.5%). Seventeen studies (n = 3285) reported recurrence following initially successful ED, with a pooled rate of 33.9% (95% CI: 19.5–52.1%; I^2^ = 97.5%). Sixteen studies (n = 2790) reported mortality; the pooled estimate was 22.6% (95% CI: 18.7–26.4%; I^2^ = 99.6%). This extreme heterogeneity likely reflects variations in patient comorbidities (case-mix) and differing outcome reporting windows rather than procedural risk in isolation. **Conclusions:** ED is an effective first-line stabilizing intervention for uncomplicated sigmoid volvulus; however, recurrence rates remain high, and outcome estimates exhibit significant heterogeneity. ED should be integrated within a structured clinical pathway that prioritizes standardized mucosal assessment, post-procedural decompression, and the timely planning of definitive management when feasible.

## 1. Introduction

Sigmoid volvulus (SV) represents a critical surgical emergency characterized by the torsion of the sigmoid colon around its mesenteric axis, leading to a closed-loop obstruction that can rapidly progress to bowel ischemia, gangrene, and perforation. While SV accounts for only 2–5% of large bowel obstructions in Western countries, its incidence dramatically increases up to 50% in the “volvulus belt” encompassing parts of Africa, Asia, and the Middle East [1,2]. Recent epidemiological data from the United States suggest that while the incidence of SV remains stable, it disproportionately affects elderly males with significant neuropsychiatric comorbidities and chronic constipation [3].

The pathophysiology of SV is inherently linked to a combination of anatomical predisposition—specifically a redundant sigmoid colon with a narrow mesenteric base—and chronic non-anatomical drivers [4]. Factors such as high-fiber diets, chronic laxative use, and refractory constipation are well-recognized contributors to the progressive colonic elongation and dilation that facilitate torsion [5,6]. Given the high baseline risk in these often frail, geriatric populations, timely diagnosis and intervention are paramount to prevent catastrophic complications, as gangrenous SV is associated with a doubling of the mortality rate compared to uncomplicated cases [1,6].

Current clinical practice guidelines from the World Society of Emergency Surgery (WSES) [7], the American Society of Colon and Rectal Surgeons (ASCRS) [8], and the American Society for Gastrointestinal Endoscopy (ASGE) [9] consistently advocate for endoscopic detorsion (ED) as the primary stabilizing intervention for patients without clinical signs of peritonitis or bowel necrosis [7,10,11]. ED offers several critical advantages: it facilitates rapid physiological decompression, permits direct assessment of mucosal viability, and creates a strategic therapeutic window to optimize the patient for definitive treatment [7,12].

However, the management of SV has undergone a significant temporal evolution. The transition from rigid to flexible endoscopy has markedly improved technical success rates and safety profiles [12,13]. Despite these advancements, post-procedural recurrence remains a formidable challenge, with recent multicenter data indicating that conservative management alone is associated with high recurrence rates and increased long-term mortality [14]. Consequently, current expert consensus emphasizes a sequential approach: initial endoscopic stabilization followed by elective sigmoid resection during the same hospital admission to mitigate the risks of recurrent emergencies [11,13].

Despite the broad endorsement of ED, substantial variability persists in reported clinical outcomes, success rates, and management protocols. This systematic review and meta-analysis aim to synthesize the evidence on the efficacy and safety of ED, evaluate the predictors of recurrence, and propose an updated clinical algorithm to refine the management of this time-sensitive surgical emergency.

## 2. Materials and Methods

### 2.1. Study Design

We conducted a systematic review and meta-analysis to systematically evaluate the diagnostic and therapeutic approaches for sigmoid volvulus, with a specific focus on endoscopic detorsion (ED) and perioperative management. The primary objective was to assess the therapeutic efficacy and safety profiles of endoscopic and surgical strategies by synthesizing clinical outcomes, recurrence rates, and mortality data. Secondary objectives included identifying prognostic predictors of recurrence and proposing an updated management algorithm. The methodology was predefined in accordance with the Preferred Reporting Items for Systematic Reviews and Meta-Analyses (PRISMA of Supplementary Materials) guidelines. The study protocol was registered prospectively with PROSPERO (ID: 1273790).

### 2.2. Eligibility Criteria

**Inclusion Criteria.** Eligible studies included original prospective and retrospective cohorts reporting outcomes following endoscopic detorsion (ED) for sigmoid volvulus. To be included, studies were required to provide data on at least one of the following endpoints: technical success, post-ED recurrence, or mortality (either in-hospital or at predefined follow-up intervals). Only studies with a sample size of at least five patients (n ≥ 5) were considered for inclusion.

**Exclusion Criteria.** Excluded publications comprised literature reviews, letters to the editor, and single case reports. Studies relying exclusively on administrative discharge databases that lacked sufficient clinical granularity (specifically regarding patient denominators, follow-up time windows, or treatment subgroups) were excluded from the quantitative synthesis and were summarized descriptively where appropriate. Furthermore, studies were excluded if ED outcomes could not be clearly distinguished from the results of other therapeutic strategies. Studies lacking data for specific secondary outcomes were nonetheless retained in the systematic review and analyzed for the specific endpoints they reported.

### 2.3. Search Strategy and Study Selection

A comprehensive and systematic literature search was executed on PubMed/MEDLINE and adapted for equivalent platforms, including Ovid/MEDLINE and Embase, utilizing a combination of MeSH terms, Emtree terms, and free-text keywords (targeting the title/abstract). To identify potentially relevant series not yet indexed, we performed hand-searching of reference lists from eligible articles, proceedings from surgical and endoscopic society meetings, and clinical study registries to capture gray literature. The search encompassed all records from database inception through 20 October 2025. Search terms targeted the condition (“sigmoid volvulus”, “colonic volvulus”) and the intervention (“endoscopic detorsion”, “endoscopic decompression”, or “endoscopy”, including historical synonyms such as “rigid sigmoidoscopy”), employing Boolean operators (AND/OR) and MeSH/EMTREE mapping where applicable. No initial language or date-restrictive filters were applied to ensure a broad capture of the literature; methodological screening was performed during the subsequent study selection phase.

Records were deduplicated using reference-management software. Two reviewers independently screened titles, abstracts, and full texts against the pre-specified eligibility criteria. Any discrepancies were resolved through consensus or via arbitration with a third senior reviewer.

### 2.4. Data Extraction and Quality Assessment

Data extraction was performed using a standardized electronic form. For each included study, we extracted the following variables where available: author and year of publication, country/setting, study design (prospective vs. retrospective; single-center vs. multicenter), total number of sigmoid volvulus cases (n), and the specific ED subgroup. Additionally, we recorded the study period, patient demographics (age, sex, comorbidities), eligibility criteria for ED, endoscopic mucosal findings, and ED modality (flexible endoscopy, rigid rectosigmoidoscopy, or adjunctive use of a rectal decompression tube). Outcomes of interest included technical success, recurrence, complications, reinterventions (including emergency or elective surgery), length of hospital stay, follow-up duration, type of subsequent surgery, and mortality (in-hospital or at defined follow-up intervals).

When studies reported outcomes exclusively as percentages without providing raw data, absolute counts were back-calculated using the most appropriate denominator: preferentially, the ED-treated population for technical success and recurrence, and the entire cohort as the denominator when mortality was not stratified by treatment modality.

**Figure 1:**
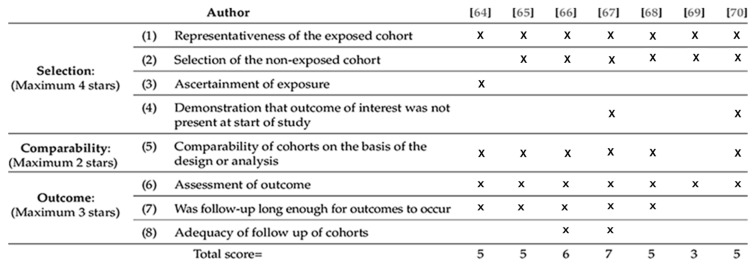
Quality Assessment.

Given the heterogeneous nature of the reporting, a single risk-of-bias tool could not be applied uniformly across all outcomes. The methodological quality of the included observational studies was systematically appraised using the Newcastle–Ottawa Scale (NOS), which evaluates three primary domains: Selection, Comparability, and Outcome/Follow-up (total score range: 0–9) (Figure 1).

### 2.5. Outcomes

#### 2.5.1. Primary Outcome

– **Technical success of endoscopic detorsion (ED)** was defined as the successful reduction in the sigmoid torsion with the subsequent restoration of colonic transit and clinical or radiological decompression following colonoscopy or flexible/rigid sigmoidoscopy. Technical success was defined as the advancement of the endoscope beyond the site of torsion, resulting in the immediate evacuation of gas or feces and a marked reduction in abdominal distension. Post-procedural clinical resolution and/or radiological evidence of detorsion were utilized as confirmatory indicators of success. Cases in which ED alone, or ED followed by the placement of a rectal decompression tube or catheter, precluded the need for immediate surgery were classified as successful. Conversely, incomplete detorsion, the need for urgent laparotomy due to impending ischemia or perforation, or overall procedural failure were classified as technical failures. When studies reported technical success exclusively as a percentage, numerators were back-calculated using the number of attempted ED procedures as the denominator; all assumptions utilized for this reconstruction are documented in the data extraction sheet.

#### 2.5.2. Secondary Outcomes

– **Recurrence following initially successful ED** was defined as a subsequent documented episode of sigmoid volvulus. When reported, recurrences were categorized as early (occurring during the index admission or within 30 days) or late (occurring after 30 days). In cases where the timing was not specified by the authors, any radiographically or endoscopically confirmed recurrence following initial success was recorded. For meta-analytical purposes, recurrence rates were calculated using the population of patients who achieved initial technical success as the denominator, thereby excluding primary technical failures.
– **Mortality** was defined as all-cause death related to sigmoid volvulus or to subsequent endoscopic or surgical interventions within the time window reported by the study authors. When mortality was not stratified specifically by treatment modality, the overall cohort mortality was utilized. Where data permitted, mortality was stratified by the reported time window, including in-hospital, 30-day, 90-day, and long-term mortality. This endpoint encompassed deaths directly attributable to bowel ischemia or perforation, as well as those secondary to patient comorbidities or perioperative complications.

### 2.6. Statistical Analysis

Pooled proportions were synthesized using a DerSimonian–Laird random-effects model on the logit scale, applying a continuity correction for rare or zero events. Statistical heterogeneity among the included studies was quantified using the Cochran’s Q statistic. Studies based exclusively on administrative databases were excluded from the formal meta-analysis and summarized descriptively to maintain clinical granularity in the pooled estimates.

## 3. Results

### 3.1. Study Identification

The systematic literature search yielded 890 records. Following the removal of 167 duplicates, 723 records remained for further evaluation. Ineligible records excluded by automation tools accounted for 458 entries, leaving 265 for title and abstract screening. During this stage, 230 records were excluded as they did not meet the predefined inclusion criteria.

**Figure 2:**
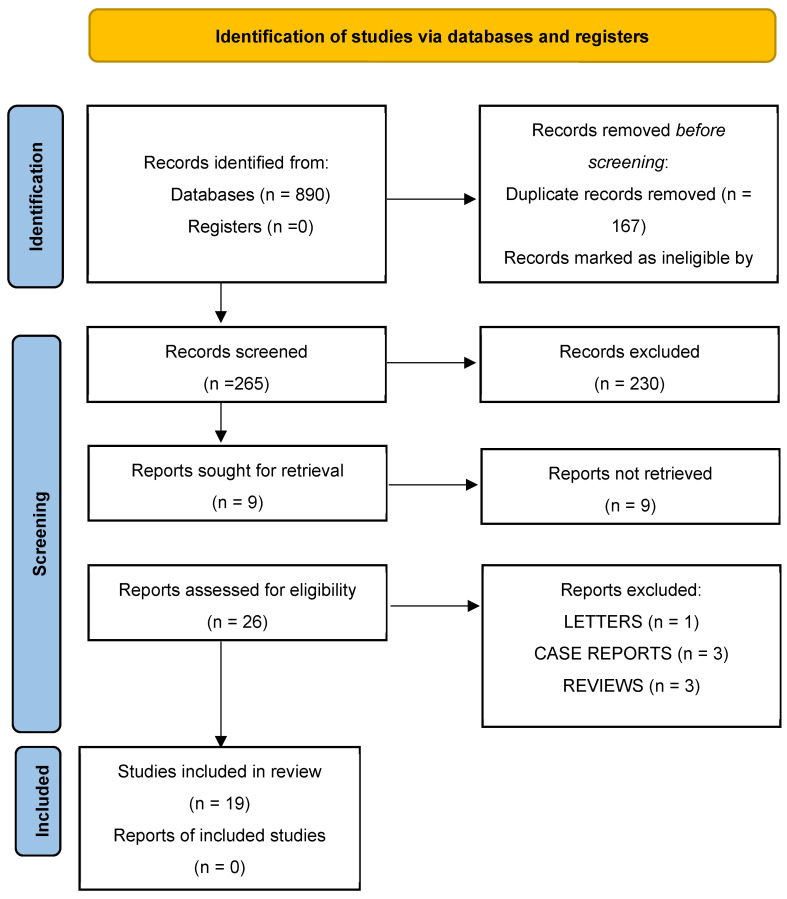
Prisma flowchart.

Of the remaining 35 reports sought for full-text assessment, 9 could not be retrieved. The remaining 26 full-text articles were critically assessed for eligibility. Following this review, seven reports were excluded for the following reasons: one letter to the editor, three case reports with a sample size of fewer than five patients (n < 5), and three review articles. Ultimately, 19 studies met all inclusion criteria and were included in both the qualitative and quantitative syntheses (Figure 2).

### 3.2. Characteristics of Included Studies

The 19 included studies were published between 2004 and 2025, with a predominance of retrospective cohorts and case series conducted in single-center settings (Table 1).

### 3.3. Patient Characteristics

The included studies describe a clinically consistent population characteristic of uncomplicated sigmoid volvulus. Mean or median age values across series ranged from 58.6 to 82.0 years, with most studies reporting mean values above 70 years, confirming a predominantly geriatric cohort with a male predominance (male-to-female ratio of approximately 2:1). Frailty and multimorbidity were prevalent; neuropsychiatric disorders were frequent (ranging from 25–67%), and chronic constipation was highly prevalent in several cohorts (up to 84.8%). Furthermore, many patients were institutionalized or experienced impaired functional autonomy.

Objective indices confirmed the high baseline clinical risk: in some series, more than half of the patients had a Charlson Comorbidity Index (CCI) > 6, and ASA III–IV scores were frequently reported. Several studies also documented markers of systemic inflammation at admission (e.g., fever, leukocytosis), widespread laxative use as a proxy for refractory constipation, and a notable prevalence of prior abdominal surgery, which may contribute to volvulus through anatomical alterations or adhesive disease.

Inclusion criteria for endoscopic detorsion were largely consistent: all studies required uncomplicated disease, defined by the absence of ischemia, peritonitis, or perforation. Some authors specified additional exclusion criteria based on clinical, endoscopic, or radiological suspicion of advanced ischemia, whereas others utilized pragmatic assessments of physiological stability. Selected series applied formal risk stratification (ASA, CCI) to refine indications but did not extend ED to complicated cases.

In summary, the evidence portrays a uniform clinical phenotype: elderly, frail patients with substantial comorbidities, frequent neuropsychiatric disease, institutionalization, and chronic constipation. Endoscopic detorsion was consistently applied to uncomplicated sigmoid volvulus, while it was systematically excluded in patients presenting with ischemia and peritonitis (Table 2).

### 3.4. Characteristics of Volvulus and Recurrent Patients

Recurrent sigmoid volvulus arises from a complex interaction between marked anatomic abnormalities, patient frailty, and non-anatomic drivers of care. Imaging reveals a distinct phenotype in early recurrences characterized by organo-axial torsion, pronounced colonic distension (mean diameter ≈ 10.0 cm; with >40% measuring >10 cm), and more frequent radiological signs of severity (e.g., torsion knot, reduced wall enhancement, bowel-wall thinning ≈ 26%, and mesenteric fluid ≈ 53%); some series also report reduced left-lobe liver volume. In contrast, patients without recurrence exhibit milder findings (mean diameter ≈ 8.8 cm; 19% > 10 cm) and larger left lateral liver volumes [18].

Clinically, patients with recurrence are older (mean age ≈ 78 years), predominantly male, frequently institutionalized, cognitively or neurologically impaired, and functionally dependent; approximately 24% have a history of prior abdominal surgery [22]. These factors collectively diminish physiological reserve and increase recurrence risk. Large registry data reveal significant demographic differences in management (e.g., Hispanic and African American patients more often receive repeat endoscopic detorsion, whereas Caucasian patients more often undergo surgery), highlighting the influence of healthcare access and practice patterns [13].

### 3.5. Characteristics of the Endoscopic Technique

Flexible endoscopy (flexible sigmoidoscopy or colonoscopy) represents the current gold standard for endoscopic detorsion (ED), whereas rigid techniques are now largely considered obsolete. The placement of post-detorsion rectal decompression tubes [4,13,14,15,19] is a common practice, with a typical duration of 12–72 h; however, its utilization is inconsistently reported across the literature. Clinical protocols vary significantly: some centers routinely utilize decompression tubes or reserve surgical intervention only for ED failure, ischemia, or perforation, whereas others integrate ED with endoscopic fixation techniques or scheduled elective surgery [5,12,18,20,23]. Administrative datasets confirm the widespread implementation of sigmoidoscopy and colonoscopy but often lack granular procedural details regarding the specific techniques and adjuncts employed (Table 3) [16,21,22,24].

### 3.6. Assessment of the Quality of Included Studies

Most included studies were single-center retrospective series of moderate quality (Newcastle–Ottawa Scale 3–7/9), with older or incomplete reports scoring lower and more recent, adjusted studies scoring higher (Table 4)

– Selection: adequate (2–3 stars) for defining populations and confirming diagnoses; historical series were less representative.
– Comparability: the main weakness; few studies adjusted for key confounders (age, comorbidity, severity).
– Outcome and follow-up: heterogeneous; studies with ≥6 months follow-up or systematic recurrence assessment scored better, while many reported only in-hospital outcomes or omitted loss to follow-up. Administrative databases showed a broad selection but limited clinical detail.

**Table 4:** Assessment of the Quality of Included Studies.

Author	Kind of Study	Selection (0–4)	Comparability (0–2)	Outcome (0–3)	Total/9
Firat et al. [15]	Retrospective observational	3	1	2	6
Atamanalp [14]	Retrospective observational	2	0	1	3
Quénéhervé et al. [13]	Retrospective observational	3	1	2	6
Moro-Valdezate et al. [17]	Retrospective observational	3	2	2	7
Turan et al. [4]	Retrospective observational	2	0	1	3
Nakamatsu et al. [19]	Retrospective observational	3	1	1	5
Iida et al. [12]	Retrospective observational	3	1	1	5
Humbert et al. [18]	Retrospective observational	3	1	2	6
Rafaqat et al. [23]	National administrative database	3	1	1	5
Tan et al. [5]	Retrospective observational	3	1	1	5
Aksungur et al. [24]	Retrospective observational	2	0	1	3
Negm et al. [20]	Retrospective observational	3	1	1	5
Abdelrahim et al. [16]	Retrospective observational	3	1	1	5
Dahiya et al. [22]	National administrative database	3	1	1	5
Korkut et al. [21]	Retrospective observational	2	0	1	3
Aminov et al. [26]	Retrospective observational	3	1	1	5
Yassaie et al. [6]	Retrospective observational	3	1	1	5
Yoen Namgung et al. [25]	Retrospective observational	3	1	1	5
Tantinam et al. [27]	Retrospective observational	2	0	1	3

### 3.7. Results of the Meta-Analysis

#### 3.7.1. Effectiveness of Endoscopic Detorsion (ED)

Fifteen clinical studies published between 2004 and 2025 provided extractable data regarding attempted endoscopic detorsion (ED) (Turan et al. [4], 2004; Tan et al. [5], 2010; Yassaie et al. [6], 2013; Atamanalp [14], 2013; Iida et al. [12], 2017; Quénéhervé et al. [13], 2019; Fırat et al. [15], 2020; Abdelrahim et al. [16], 2021; Moro-Valdezate et al. [17], 2022; Nakamatsu et al. [19], 2023; Negm et al. [20], 2023; Aksungur et al. [24], 2024; Namgung et al. [25], 2024; Aminov et al. [26], 2025; Tantinam et al. [27], 2025), comprising a cumulative cohort of 1738 patients. Collectively, these reports encompass clinical practice from 1966 to 2024, offering a comprehensive longitudinal perspective on the evolution of ED across various geographical regions and eras. Four studies were excluded from the quantitative synthesis as they did not provide granular or verifiable data on technical success (Humbert [18], Dahiya [22], Rafaqat [23], and Korkut [21]).

Technical success was operationally defined as the complete resolution of torsion with effective colonic decompression and restoration of bowel patency during the index endoscopic procedure, thereby obviating the requirement for immediate surgical intervention. This outcome was typically documented by the passage of the endoscope beyond the torsion point, the evacuation of gas or fecal material, and a direct endoscopic assessment of mucosal integrity. While additional study-specific criteria were recorded in the data extraction table, they did not alter the common operational definition established for the quantitative synthesis (Table 5).

**Figure 3:**
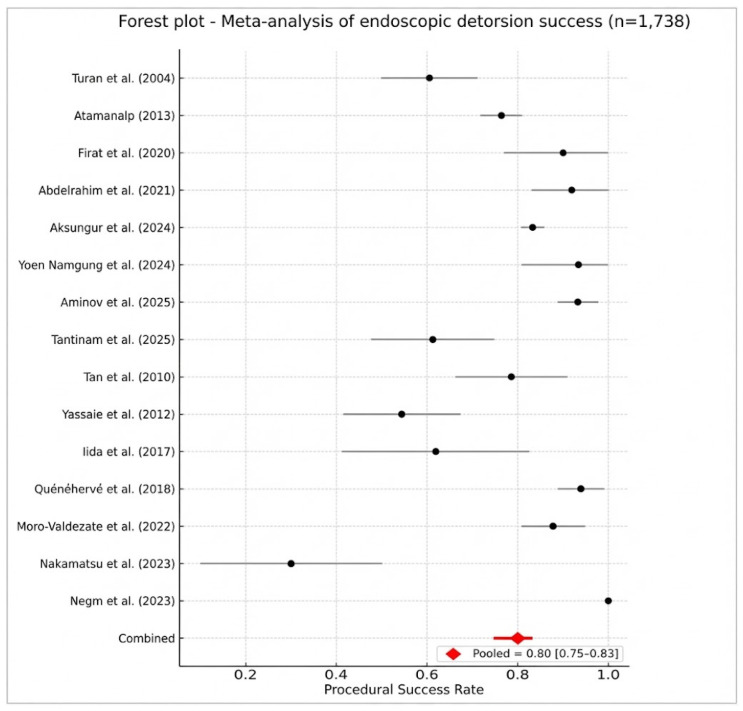
Success rate (forest plot) [4,5,6,12,13,14,15,16,17,19,20,24,25,26,27].

Reported per-study success rates exhibited significant variability, reflecting disparities in patient selection, timing of intervention, operator experience, endoscopic modalities, and the specific study eras. Technical success was pooled using a logit transformation and a DerSimonian–Laird random-effects model, yielding an overall pooled success rate of 80.0% (95% CI: 75.0–83.0%). Between-study heterogeneity was statistically significant and high (I^2^ = 87.5%; τ^2^ = 0.232), indicating substantial clinical and methodological variability across the included cohorts (Figure 3).

Reported technical success rates for endoscopic detorsion (ED) ranged from less than 30% to approximately 100%. Lower success rates were more prevalent in historical series or in clinical settings with limited access to flexible endoscopy; representative examples include Turan et al. [4] (60%), Yassaie et al. [6] (54%), Iida et al. [12] (62%), and Nakamatsu et al. [19] (28.6%).

In contrast, contemporary high-volume expertise centers reported consistently superior outcomes: Quénéhervé et al. [13] (94%), Fırat et al. [15] (90%), Abdelrahim et al. [16] (92.5%), Moro-Valdezate et al. [17] (87.8%), Namgung et al. [25] (93.3%), and Aminov et al. [26] (93.5%). Intermediate results were documented in large-scale, multi-year cohorts, such as those by Atamanalp [14] (76.4%) and Aksungur et al. [24] (83.2%), while a 100% success rate was achieved in the highly selective series by Negm et al. [20]. These findings indicate that while ED demonstrates a high probability of immediate success in modern centers utilizing routine flexible endoscopy, clinical outcomes remain highly context-dependent—fundamentally shaped by patient selection, procedural timing, operative technique, and operator experience.

**Figure 4:**
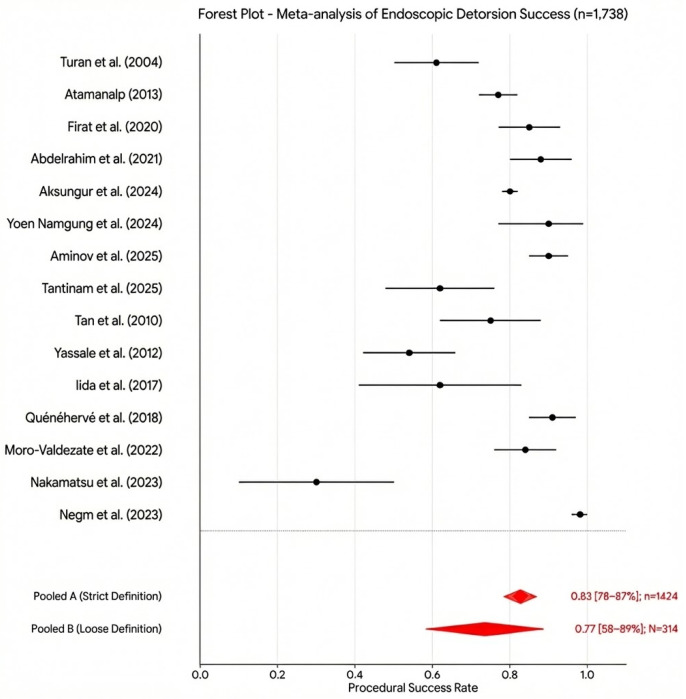
Definition of endoscopic success (forest plot) [4,5,6,12,13,14,15,16,17,19,20,24,25,26,27].

To further investigate this variability, we stratified the literature according to the operational definition of “success,” identifying two distinct clinical patterns. In the first subgroup, where success was defined strictly by successful endoscopic passage, direct visualization of viable mucosa, and avoidance of immediate surgical intervention, the pooled success rate was 83% (95% CI: 78–87%; n = 1424), although substantial inter-center variability persisted. In the second subgroup, where success was judged solely by symptomatic decompression, the pooled rate decreased to 77% (95% CI: 58–89%; N = 314), with a wider dispersion of results reflecting inconsistent clinical thresholds and follow-up protocols (Figure 4).

The discrepancy between these two analytical strands underscores a critical methodological point: the definition of success fundamentally dictates the clinical narrative. Studies mandating systematic mucosal assessment typically originate from institutions with standardized protocols and routine flexible endoscopy; these report marginally higher success rates, yet both groups continue to exhibit significant statistical heterogeneity. The practical implication is both clear and urgent: to ensure that future reports are comparable and clinically actionable, a single, transparent definition of endoscopic success is required—one that integrates technical traversal and decompression with an explicit assessment of mucosal viability. Furthermore, routine reporting of subgroup analyses and the I^2^ statistic is essential to fully elucidate the texture of the evidence.

#### 3.7.2. Recurrence of Sigmoid Volvulus After Endoscopic Derotation (ED)

**Figure 5:**
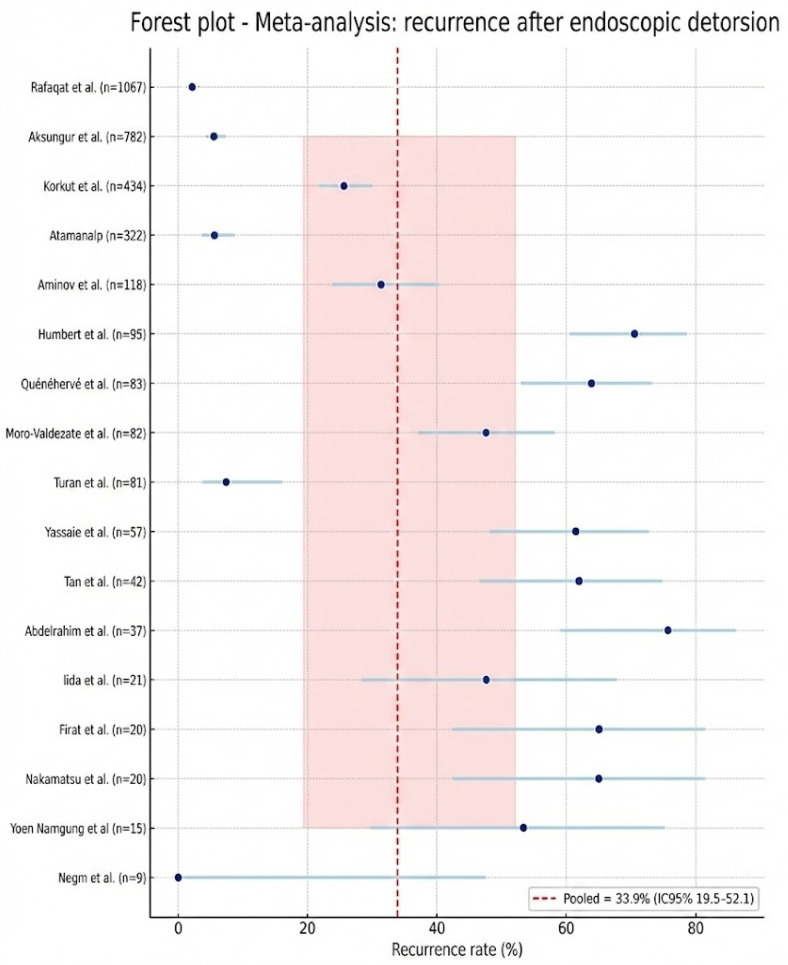
Recurrence after endoscopic detorsion: Meta-analysis [4,5,6,12,13,14,15,16,17,18,19,20,21,23,24,25,26].

Data regarding recurrence following successful endoscopic detorsion were extractable from seventeen studies [4,5,6,12,13,14,15,16,17,18,19,20,21,23,24,25,26], encompassing a total of 3285 patients (Figure 5). The pooled recurrence rate was 33.9% (95% CI: 19.5–52.1%). Significant statistical heterogeneity was observed (I^2^ = 97.5%), indicating substantial clinical and methodological divergence across the included cohorts, particularly concerning post-procedural surveillance and definitive management strategies.

The observed variability correlated with several identifiable factors. Studies utilizing longer or more comprehensive follow-up periods reported significantly higher recurrence rates. Conversely, cohorts that routinely performed elective sigmoid resection or endoscopic fixation after the index ED reported a marked reduction in recurrent episodes compared to those managed expectantly. Key prognostic determinants, including advanced age, frailty, institutionalization, chronic constipation, and a history of abdominal surgery, were associated with an increased risk of recurrence. While technical and procedural factors (such as the routine use of flexible endoscopy, rectal tube placement, and operator experience) reduced early procedural failure, they did not demonstrate a consistent impact on long-term recurrence rates. Additionally, the data source influenced the outcomes: administrative database records frequently yielded different recurrence estimates compared to prospective clinical follow-up cohorts.

To account for this heterogeneity, analyses were stratified by follow-up duration (<30 days vs. >30 days), the implementation of definitive treatment post-ED, and study quality. These subgroup analyses revealed significantly lower recurrence rates in cohorts with short-term follow-up or routine definitive management. While recurrence after ED is common and clinically significant, the pooled proportion must be interpreted in the context of follow-up duration, local practice patterns, and patient-specific comorbidities.

Moving forward, it is essential to standardize outcome reporting by utilizing time-to-event analyses (e.g., Kaplan–Meier estimates or cumulative incidence at 30, 90, and 365 days). Furthermore, clear definitions of recurrence (whether clinical, radiologic, or endoscopic) and explicit documentation of follow-up windows and censoring rules are imperative to ensure the clinical utility and comparability of pooled estimates.

#### 3.7.3. Mortality in Patients with Sigmoid Volvulus After Endoscopic Detorsion (ED)

Evidence regarding mortality was derived from 16 clinical studies [4,5,6,12,13,14,15,16,17,20,22,23,24,25,26,27], comprising 2790 patients, and was complemented by two administrative datasets incorporating 222,799 additional cases. Due to substantial differences in granularity and outcome ascertainment between clinical cohorts and administrative records, these data sources were analyzed separately to maintain methodological integrity.

Clinical reports demonstrate that mortality is critically influenced by the clinical scenario. Endoscopic detorsion (ED) for uncomplicated cases in contemporary high-expertise centers is associated with low mortality rates, typically ranging from less than 1% to 6%. Conversely, the presence of bowel ischemia, perforation, or the requirement for emergency surgical intervention substantially increases the mortality risk, with operative mortality often reaching 15–25%. Elective surgery following successful stabilization is associated with favorable perioperative outcomes, with mortality rates ranging between 0% and 9%.

Disparities in reported mortality reflect differences in patient demographics (age, comorbidities, and institutionalization), treatment allocation (primary endoscopy versus emergency laparotomy), and the defined observation period (in-hospital, 30-day, or long-term follow-up). Administrative databases, while encompassing vast populations, lack the clinical granularity required to reliably attribute the cause of death; consequently, these were analyzed descriptively rather than pooled with clinical series.

**Figure 6:**
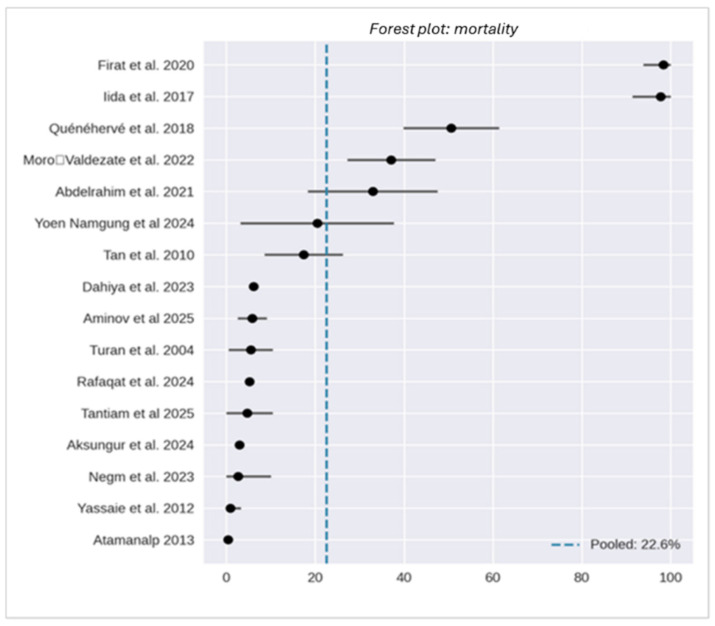
Mortality (forest plot) [4,5,6,12,13,14,15,16,17,20,22,23,24,25,26,27].

Overall, endoscopic management of uncomplicated sigmoid volvulus demonstrates a favorable safety profile in modern practice; however, patient frailty and the necessity for emergency operative care remain the primary drivers of mortality. Notably, conservative management was associated with an extremely high risk in frail populations, with reported rates up to 70%. The pooled mortality estimate was 22.6% (95% CI: 18.7–26.4%), with extreme heterogeneity (I^2^ = 99.6%). This high I^2^ value reflects the diversity of the included populations and follow-up windows rather than an intrinsic hazard of the endoscopic procedure itself (Figure 6).

To enhance the clinical utility and comparability of future mortality estimates, research should prioritize the standardization of outcome windows, the use of time-to-event analyses, and the explicit clarification of treatment allocation strategies.

## 4. Discussion

This systematic review offers an integrated and comprehensive synthesis of decades of clinical experience regarding endoscopic detorsion (ED) for sigmoid volvulus and the subsequent therapeutic pathway, aligning our findings with the current recommendations of leading surgical and endoscopic societies. The analysis clearly demonstrates that ED, when performed in selected patients and in a timely manner, resolves the acute event in the majority of cases; immediate decompression and the inherent capacity to directly evaluate mucosal viability underlie the primary role that guidelines [7,8,9] assign to this procedure in uncomplicated cases. This concordance between aggregated evidence and guideline-oriented indications is noteworthy: when detorsion is performed via flexible endoscopy by expert operators using protocols that mandate mucosal verification and post-procedural decompression, technical success rates are high and periprocedural morbidity remains limited.

Addressing the variability of results, however, requires a nuanced interpretation. The data show a notable dispersion in success, recurrence, and mortality rates that is not attributable to statistical error but to real-world clinical heterogeneity: the chronological era of the studies, the technology employed, patient selection criteria, varying definitions of “success” and “recurrence,” the expertise level of the centers, and the follow-up duration. Consequently, interpretation must be contextual. Historical series and those conducted with rigid instruments record less favorable results, whereas contemporary cohorts—where flexible endoscopy is available, and procedures are performed according to consolidated protocols—show significantly superior performance. The necessity of verifying mucosal viability at the end of the maneuver, as mandated by international guidelines [7,8,9], finds empirical confirmation in our data: endoscopic documentation of the mucosa is the pivotal determinant that determines whether to proceed with stabilization or transfer the patient directly to the operating suite, and adherence to this practice reduces both immediate failures and exposure to severe complications.

Post-procedural management represents another pivotal clinical step. The practice of placing a rectal tube or flatus tube and maintaining decompression for 24–72 h is recommended and, in datasets where it has been systematically applied, a reduction in early recurrences is observed. This confirms the pragmatic value of simple, low-cost measures that, when implemented in standardized pathways, improve clinical stability in the short term. Guidelines [7,8,9] emphasize and justify this strategy: the tube is not a technical adjunct but an important tool for preventing immediate torsion recurrence, and the evidence collected reinforces this recommendation with concrete data. Simultaneously, indications against the administration of oral bowel preparations in the acute phase remain well-founded and consistent with the principle of avoiding exacerbation of colonic distension.

The issue of recurrence is perhaps the factor that most influences therapeutic reasoning and creates the greatest gap between current evidence and optimal practice. This meta-analysis synthesizes a cumulative risk of approximately one-third of patients; however, this figure encompasses both early recurrences linked to technical failure or inadequate stabilization and late recurrences that result from an underlying anatomical predisposition (e.g., dolichosigmoid, hypermobile mesocolon, mesenteric laxity) that endoscopy alone cannot resolve. For this reason, whenever feasible, elective sigmoid resection following successful ED-mediated stabilization should be performed. The rationale is that transforming temporary resolution into a definitive solution reduces the risk of future emergencies, which are associated with significantly higher mortality and morbidity compared to scheduled interventions. Data from series adopting a sequential approach (detorsion for stabilization followed by elective resection) show a more favorable overall risk profile compared to prolonged expectant management or emergent conversion following recurrence.

Mortality must be interpreted in light of cohort composition and therapeutic timing. The aggregate of studies and databases produces an elevated overall estimate when considering the entire population of patients with volvulus; however, stratified analysis reveals that mortality directly attributable to ED in uncomplicated patients is very low. Mortality increases drastically when the patient presents with ischemia, perforation, or shock, and when surgery is performed emergently; in these scenarios, timely resection remains the primary life-saving measure. The correlation between frailty (advanced age, high comorbidity index, ASA status) and outcome is strong and consistent: in studies stratifying by ASA or comorbidity index, mortality varies markedly as a function of patient frailty rather than the specific technique chosen, highlighting the need for an early multidisciplinary approach—including anesthesiology and geriatrics—to optimize patient selection and the timing of interventions.

A recurrent methodological aspect is the lack of uniformity in definitions and the variability of follow-up. Guidelines [7,8,9] draw indications from heterogeneous literature, and as emerged from this review, operative differences in the definitions of “success” or “recurrence” significantly influence estimates. This limits the ability to directly translate aggregate percentages into practical recommendations and underscores the urgent need for standardized endpoints in future research. Similarly, large administrative databases provide extensive numbers but lack the clinical granularity necessary to distinguish periprocedural mortality from long-term outcomes or to verify the actual execution of the endoscopic maneuver, a phenomenon that mandates caution in interpreting published results.

Comparison with international guidelines [7,8,9] highlights a substantial alignment on principles and priorities: ED as the first line in uncomplicated forms, mandatory mucosal viability verification, placement of a decompression tube, and the recommendation to plan, whenever feasible, an elective resection to reduce future recurrences and emergencies. Areas of greater uncertainty concern operative details that the literature has not yet definitively established: the optimal timing of elective resection across the spectrum of frail patients, the precise identification of subjects for whom endoscopic fixation or percutaneous endoscopic colopexy (PEC) is an acceptable option, and the long-term impact on quality of life across different strategies. These gaps represent a priority for research: prospective multicenter studies with unified endpoints and the systematic collection of prognostic variables (e.g., colonic diameter, symptom duration, torsion angle, presence of ileosigmoid knot, tube use) are necessary to transform statistical power into precise, transferable clinical recommendations.

In daily clinical practice, the operative message derived from the integration of evidence and guidelines [7,8,9] is pragmatic in nature. ED remains the tool that allows for the avoidance of emergent intervention in many patients and creates a useful therapeutic window for an informed surgical decision; its technical efficacy and safety profile in uncomplicated cases make it the rational initial choice. Simultaneously, awareness of recurrence risks and the significant influence of frailty on mortality indicates that detorsion should not be viewed as a definitive endpoint but as a stage in a therapeutic pathway requiring multidisciplinary evaluation and the planning of definitive treatment. Implementing local protocols that standardize selection, periprocedural management, discharge criteria, and follow-up is the most concrete path toward reducing outcome variability and aligning daily practice with the best available evidence.

## 5. Conclusions

In conclusion, endoscopic detorsion (ED) constitutes a safe and effective first-line intervention for the management of acute, uncomplicated sigmoid volvulus, demonstrating a pooled technical success rate of 80.0%. The therapeutic utility of ED is fundamentally derived from its capacity to facilitate rapid physiological decompression, provide a direct assessment of mucosal viability, and stabilize the patient, thereby creating a pivotal therapeutic window to facilitate the planning of definitive management. Our findings demonstrate that while ED is highly effective in modern centers utilizing flexible endoscopy and standardized protocols, clinical outcomes remain context-dependent and are significantly influenced by procedural timing and anatomical predictors, notably colonic distension exceeding 10 cm.

Recurrence remains a substantial challenge, with a pooled rate of approximately 34%. This risk is primarily driven by underlying anatomical predispositions and patient frailty rather than procedural failure, reinforcing the role of ED as a stabilizing bridge to definitive therapy rather than a curative modality in isolation. Furthermore, the mortality associated with sigmoid volvulus is predominantly determined by the initial presentation and patient comorbidities—specifically ASA III–IV status—rather than the endoscopic procedure itself, which maintains a favorable safety profile in uncomplicated cases. Consequently, ED should be integrated into a structured multidisciplinary clinical pathway that prioritizes mandatory post-procedural decompression via rectal tubes and timely elective sigmoidectomy to mitigate the risks of recurrent emergencies and the associated high morbidity of urgent re-interventions. Future research must focus on the standardization of outcome definitions and the implementation of prospective multicenter trials to refine prognostic algorithms and optimize long-term survival in this time-sensitive surgical emergency.

## Figures and Tables

**Table 1:** Included studies.

Author	Year	Kind of Study
Turan et al. [4]	2004	Retrospective Observational Study
Tan et al. [5]	2010	Retrospective Observational Study
Yassaie et al. [6]	2012	Retrospective Observational Study
Atamanalp [14]	2013	Retrospective Observational Study
Iida et al. [12]	2017	Retrospective Observational Study
Quénéhervé et al. [13]	2018	Retrospective Observational Study
Firat et al. [15]	2020	Retrospective Observational Study
Abdelrahim et al. [16]	2021	Retrospective Observational Study
Moro-Valdezate et al. [17]	2022	Retrospective Observational Study
Humbert et al. [18]	2022	Retrospective Observational Study
Nakamatsu et al. [19]	2023	Retrospective Observational Study
Negm et al. [20]	2023	Retrospective Observational Study
Korkut et al. [21]	2023	Retrospective Observational Study
Dahiya et al. [22]	2023	Retrospective Observational Study
Rafaqat et al. [23]	2024	Retrospective Observational Study
Aksungur et al. [24]	2024	Retrospective Observational Study
Yoen Namgung et al. [25]	2024	Retrospective Observational Study
Aminov et al. [26]	2025	Retrospective Observational Study
Tantinam et al. [27]	2025	Retrospective Observational Study

**Table 2:** Patient Characteristics.

Author/Year	Median Age (Years)	Relevant Comorbidities (>15%)	Inclusion Criteria for Endoscopic Detorsion
Firat et al. [15]	61.8	25% neuropsychiatric; 15% cardiovascular	≥18 years old, absence of peritonitis/ischemia, radiologically confirmed diagnosis
Atamanalp [14]	58.6	NR	Absence of peritonitis/ischemia
Quénéhervé et al. [13]	72	48% long term institutional care; 33% loss of autonomy; 58% neurological deficit; 25% surgery	Absence of peritonitis/ischemia
Moro-Valdezate et al. [17]	81	ASA > III 20.7%; Charlson > 6 55.4%; constipation 84.8%; ipertension 58.7%; dementia 51.1%; bedridden patient 64.1%	Absence of peritonitis/gangrene
Turan et al. [4]	65	71% > 50aa; 38% > 70aa; fever/leukocytosis 95%	Absence of peritonitis/ischemia
Nakamatsu et al. [19]	75.5	63% neuropsychiatric; 51.4% constipation; 22.8% surgery	Absence of peritonitis/ischemia perforation
Iida et al. [12]	76	76% laxatives; 61.9% neurological; 57% surgery; 57% IA; 33% diabetes	Absence of peritonitis/ischemia perforation
Humbert et al. [18]	72	NR	Absence of peritonitis/ischemia perforation
Rafaqat et al. [23]	73	Frailty: low 41.6%; moderate 52.5%; high 5.9%	Absence of peritonitis/ischemia
Tan et al. [5]	73	42% ipertension; 19.7% Parkinson; 18.3% long term institutional care; 22.5% cerebrovascular	Absence of acute abdomen
Aksungur et al. [24]	59.4	NR	Non-complicated patients
Negm et al. [20]	77	16.7% diabetes; 16.7% obesity; 16.7% surgery; 22.2% recurrent colonic volvulus	Absence of peritonitis/ischemia
Abdelrahim et al. [16]	82	65% ASA III–IV; 47% neuropsychiatric	Absence of peritonitis/ischemia
Dahiya et al. [22]	66	Constipation; opioids; Parkinson; depression	Absence of peritonitis/ischemia
Korkut et al. [21]	60	elderly; dolicosigma; male; high altitude; constipation; Parkinson	Absence of peritonitis/ischemia
Aminov et al. [26]	69	40.7% diabetes; 34.7% dementia; 55.1% neurological 16.1% Parkinson	Absence of peritonitis/ischemia
Yassaie et al. [6]	68	NR	Absence of peritonitis/ischemia
Yoen Namgung et al. [25]	65	57.1% constipation; 66.7% neuropsychiatric; 38.1% long term institutional care	Absence of peritonitis/ischemia
Tantinam et al. [27]	67.5	NR	≥18 years old; Absence of peritonitis/ischemia

**Table 3:** Characteristics of Endoscopic Technique.

Author	Flexible Endoscope	Rigid Rectosigmoidoscope	Rectal Decompression Tube
Firat et al. [15]	Yes	No	No
Atamanalp [14]	Yes	No	Yes
Quénéhervé et al. [13]	Yes	No	Yes
Moro-Valdezate et al. [17]	Yes	No	Yes
Turan et al. [4]	Yes	Yes	Yes
Nakamatsu et al. [19]	Yes	No	Yes
Iida et al. [12]	Yes	No	No
Humbert et al. [18]	Yes	No	No
Rafaqat et al. [23]	Yes	No	No
Tan et al. [5]	Yes	No	Yes
Aksungur et al. [24]	Yes	No	No
Negm et al. [20]	Yes	No	No
Abdelrahim et al. [16]	Yes	No	No
Dahiya et al. [22]	Yes	No	No
Korkut et al. [21]	Yes	No	No
Aminov et al. [26]	Yes	No	No
Yassaie et al. [6]	Yes	No	Yes
Yoen Namgung et al. [25]	Yes	No	No
Tantinam et al. [27]	Yes	No	No

**Table 5:** Definition of endoscopic success for each study.

Author	Definition of Endoscopic Success
Turan et al. [4], 2004	– Complete detorsion of the sigmoid colon. – Visualization of viable mucosa (absence of ischemia or gangrene). – No need for urgent surgery.
Tan et al. [5], 2010	– Complete sigmoid decompression. – Viable mucosa at the end of the procedure. – Patient not requiring immediate surgery.
Yassaie et al. [6], 2013	– Endoscopic decompression. – Clinical resolution of obstruction. – Absence of immediate complications (ischemia, perforation).
Atamanalp [14] et al., 2013	– Complete reduction in the volvulus. – Effective colonic decompression. – Viable mucosa and post-procedural clinical stability.
Iida et al. [12], 2017	– Successful detorsion with passage beyond the point of torsion. – No signs of ischemia or perforation. – Restoration of bowel transit and clinical resolution.
Quénéhervé et al. [13], 2019	– Achievement of abdominal decompression. – Resolution of obstructive symptoms. – No indication for immediate surgery.
Firat et al. [15], 2020	– Complete detorsion and decompression of the sigmoid colon. – Viable mucosa on endoscopic inspection. – No need for urgent surgical intervention.
Abdelrahim et al. [16], 2021	– Colonic decompression. – Absence of ischemia or perforation. – No indication for emergency surgery after the procedure.
Moro-Valdezate et al. [17], 2022	– Effective endoscopic detorsion and decompression. – Absence of post-procedural complications. – No need for urgent surgery.
Negm et al. [20], 2023	– Detorsion confirmed radiologically. – Restoration of colonic lumen patency. – No immediate need for surgical intervention.
Nakamatsu et al. [19], 2023	– Endoscopic passage beyond both points of torsion. – Complete straightening and shortening of the sigmoid colon. – Decompression with gas aspiration and clinical improvement.
Aksungur et al. [24], 2024	– Complete detorsion with viable colon. – Restoration of flow and effective decompression. – No need for immediate surgery.
Namgung et al. [25], 2024	– Complete endoscopic detorsion and decompression. – Clinical resolution of obstruction. – No need for surgical intervention.
Aminov et al. [26], 2025	– Endoscopic traversal of torsion points. – Decompression of the proximal colon with gas aspiration. – Viable mucosa.
Tantinam et al. [27], 2025	– Complete detorsion of the sigmoid colon. – No need for immediate surgery. – Absence of early recurrence after the procedure.

## Data Availability

No new data were created or analyzed in this study.

## Supplementary Materials

The following supporting information can be downloaded at: https://www.mdpi.com/article/10.3390/jcm15031190/s1, PRISMA 2020 checklist [28].
